# Combination antiretroviral treatment use in prevention of mother-to-child transmission programmes: 6-week HIV prevalence and relationship to time of antiretroviral treatment initiation and mixed feeding

**DOI:** 10.4102/sajid.v34i1.117

**Published:** 2019-11-25

**Authors:** Victoria Ndarukwa, Moleen Zunza

**Affiliations:** 1 Division of Epidemiology and Biostatistics, Faculty of Medicine and Health Sciences, Stellenbosch University, Cape Town, South Africa

**Keywords:** prevention of mother-to-child transmission, HIV/AIDS, combination antiretroviral therapy, infant feeding

## Abstract

**Background:**

In Zimbabwe, 16% of pregnant women aged 15–49 years are infected with HIV. More than 90% of HIV infection in children is through mother-to-child transmission (MTCT). We investigated the effectiveness of the Option B+ in reducing HIV infection and factors associated with HIV transmission among infants born to mothers enrolled in the prevention of mother-to-child transmission (PMTCT) programme.

**Methods:**

We randomly selected 1204 early infant HIV diagnosis test results for HIV-exposed infants and linked these results to maternal clinical records at primary healthcare clinics in Harare to estimate the prevalence of MTCT and to determine the clinical factors associated with MTCT of HIV at 6 weeks.

**Results:**

Of the 1204 infants in the study, 2.5% (95% confidence interval [CI], 1.7–3.5) were infected with HIV at 6 weeks post-delivery. Antiretroviral adherence reduced the odds of HIV infection by about 99% (odds ratio [OR] 0.01 [95% CI, 0.00–0.06]). Both mixed feeding (OR 3.89 [95% CI, 0.92–16.50]) and late initiation of antiretroviral treatment (ART) (after delivery) (OR 3.18 [95% CI, 0.42–23.94]) increased the odds of HIV infection.

**Conclusion:**

Early initiation of combination ART reduces 6-week MTCT of HIV in PMTCT programmes to levels similar to those found in controlled trial settings. Exclusive breastfeeding remains important even in the presence of ART.

## Background

Zimbabwe is among the top 22 countries in the world with the highest number of pregnant women living with HIV, with an estimated HIV prevalence of 16% among women aged between 15 and 49 years.^1^ Without any interventions, women living with HIV will pass the infection to their infants during pregnancy, labour and delivery in about 15% – 25%^2^ of the cases, and an additional 5% – 20% of the infants may become HIV-infected during breastfeeding.^3^ Studies showed that when combination antiretroviral treatment (cART) is given to the mother for the duration of breastfeeding or for life, the risk is reduced further to about 5% or less.^4,5^ Many factors influence the risk of in utero transmission or post-delivery through breastfeeding. These factors include high maternal viral load, invasive obstetric procedures and suboptimal infant feeding practices.^6,7^ For example, in the first 6 months of life, the risk of mother-to-child transmission (MTCT) in infants who are given other foods in addition to breastmilk is fourfold higher than that of infants who are breastfed only.^8,9^

In 2002, when Zimbabwe launched the prevention of mother-to-child transmission (PMTCT) programme, single-dose nevirapine (Sd-NVP) was given to the mother during labour and to the infant within 3 days of birth. Women were counselled to exclusively breastfeed for the first 6 months of an infant’s life.^10^ Eight years later, following World Health Organization (WHO) guidelines which recognised that cART minimises the risk of MTCT of HIV both during pregnancy and breastfeeding,^11^ the antiretroviral treatment (ART) was changed to a combination of Zidovudine from 28 weeks of pregnancy with Sd-NVP at the onset of labour and the infant was given Sd-NVP with Zidovudine (dual treatment).^12^ The WHO have updated the ART and infant feeding guidelines three times since 2010, considering provision of cART to the mother either for the duration of breastfeeding or for life.^11,13,14^

In an effort to reduce new paediatric HIV infections over the years, Zimbabwe’s PMTCT programme has been adopting the most recent WHO recommendations on ART and infant feeding for women living with HIV. The current guidelines recommend lifelong cART for pregnant and breastfeeding women living with HIV, regardless of the CD4 count. Exclusive breastfeeding is recommended in the first 6 months of life with the introduction of complementary feeds and continued breastfeeding for 24 months or longer.^14^ Since the end of the last quarter of 2013, Zimbabwe’s Ministry of Health Care has supported the use of cART for pregnant women and lactating mothers, with a commitment to continue lifelong treatment.^15,16^ Women are started on a combination of either Tenofovir + Lamivudine + Efavirenz or Zidovudine + Lamivudine + Efavirenz, while the infants are initiated on nevirapine for 6 weeks. Women are counselled to exclusively breastfeed for the first 6 months of the infant’s life.^17^

Both small and large clinical trials and observational studies have reported MTCT rates at or below 3.5% where ART is used in pregnancy and during breastfeeding, with cART initiated prior to conception or early in pregnancy being associated with the lowest rate of MTCT.^18,19^

Since the inception of cART for PMTCT in Zimbabwe, little is known regarding HIV transmission rates at 6 weeks after birth among infants born to women living with HIV. The main aim of this study was to assess the effect of maternal cART and infant feeding practices on MTCT rates among infants born to women living with HIV in Harare, Zimbabwe. We also looked at factors that may influence the risk of HIV transmission at 6 weeks.

## Methods

### Study design

We retrospectively reviewed the early infant diagnosis National Microbiology Reference Laboratory (NMRL) registry (consisting of HIV infection test results) and the maternal clinical records of participants enrolled in the PMTCT program, over a 2-year period (March 2014 – March 2016). The study period was based on the data available after the introduction of cART in all Harare City Health polyclinics.

### Study population, setting

We included HIV-infected women participating in a PMTCT programme offered by Harare City Health polyclinics who gave birth between March 2014 and March 2016 and were receiving ongoing HIV care at the health facilities. Mother–infant dyads were eligible if the infant was a single child with a birth weight greater than or equal to 1500 g, and neither mother nor infant had an acutely life-threatening condition requiring neonatal admission and intervention. Selection of mother–infants pairs dyads was restricted to mothers who were at least 16 years of age and whose infant’s 6-week HIV infection test results were recorded in the NMRL registry. We deliberately chose all the 12 polyclinics under Harare city council for feasibility purposes. The polyclinics offer similar services which include antenatal care, PMTCT services and delivery. The majority of pregnant women attending the polyclinics are from the Harare high-density suburbs.

### Sample size

We assumed 5% of the infants would become HIV-infected 6 weeks following delivery.^4,5^ Sample size calculation was performed using STATA version14 (Stata 14 software; Stata Corp., College Station, TX, USA). Calculations were based on the power of the Wald test which we set at 0.8, with the null and alternative proportions at 0.05 and 0.0707, respectively. We set the significance level of 0.05. The *a priori* sample size calculation demonstrated that an estimated sample size of 1204 participants was needed to conduct the study.

### Study measurements and procedures

We compiled a data set on Excel of all entries from the early infant diagnosis registry at the National Microbiology laboratory comprising 5252 infants referred from the 12 Harare polyclinics and tested for HIV using polymerase chain reaction (PCR) technology between 2014 and 2016. We performed a simple random selection in Stata of 1204 out of the 5252 infants, where each infant had an equal probability of being included in the study sample. The infant was linked to the mother’s clinical records using the name of the infant, the mother’s name, the laboratory generated number and the referring clinic. The information used to link mother and infant was found at the laboratory and the polyclinic. Information with participant names was kept in the password-protected electronic file. Data were collected on forms where each participant was assigned a unique patient identifier linking to the information in the electronic file. Data were obtained by data abstraction from clinical records. The following data were retrieved from the clinical records: ART regimen taken for PMTCT, whether or not the infant was on prophylaxis, the time at which the mother initiated ART treatment, therapy adherence, counselling services offered pre- and post-partum, mode of delivery, feeding mode and employment status.

### Clinical definitions

Infants were categorised as exclusively breastfed if they receive breastmilk only without other liquids or solids, other than the use of oral rehydration solution, vitamins, minerals or medicines. Infants were categorised as recipients of exclusive replacement feeding if they never received breastmilk but were provided with commercial infant formula only without other liquids or solids, allowing for the same exceptions as noted for exclusive breastfeeding. Mixed feeding was defined as combining other liquids or solids with either breast milk or infant formula, or a combined practice breastmilk and formula feeds.^12^ Combination antiretroviral treatment adherence was defined as taking cART every day and exactly as prescribed,^20^ and the facilities measured this by quantifying missed visits and counting medicines returned.

### Statistical methods

Categorical variables were summarised as counts (per cent). Multiple logistic regression was used to determine factors that are associated with the risk of infant HIV acquisition by 6 weeks of life. Summary statistics were reported with the corresponding 95% confidence intervals (CIs). Statistical significance was set at 0.05. Statistical analyses were done using Stata version 14 (Stata 14 software; Stata Corp.).

### Ethical consideration

Ethics approval was obtained from the Human Health Research Ethics Committee of the Stellenbosch University (Reference no. S16/04/081), and from the City of Harare Ethics Committee and Medical Research Council of Zimbabwe (Reference no. MRCZ/B/1086N). Both ethics committees approved the research and waiver of consent.

## Results

We included 1204 women living with HIV and their infants in the analysis. The majority of the infants (94%) included in the study were exclusively breastfeeding in the first 6 weeks of life. Almost all women had received both pre- and post-delivery infant feeding counselling. The majority of the women were on cART, and most of them started treatment during pregnancy (see Table 1).

**TABLE 1:** Characteristics of HIV-infected women, and their infants (*n* = 1204) and risk factors associated with HIV infection.

Characteristic	*n*	%	Number of HIV positive infants	Crude odds ratio	95% CI	*p*	Adjusted odds ratio	95% CI	*p*
**Mode of delivery**									
Normal delivery	1166	97	29	1	-	-	-	-	-
Caesarean section	38	3	1	1.06	0.14–7.99	0.95	-	-	-
**Employment status**									
Yes	31	3	0	-	-	-	-	-	-
No	1173	97	29	-	-	-	-	-	-
**†Antiretroviral treatment adherence**									
No	20	2	14	1	-	-	1	-	-
Yes	1155	98	14	0.03	0.01–0.08	< 0.001	0.01	0.00–0.06	< 0.001
**†Pre-partum counselling**									
No	23	2	10	1	-	-	1	-	-
Yes	1181	98	19	0.02	0.01–0.06	< 0.001	0.65	0.6–7.14	0.72
**Post-partum counselling**									
No	10	1	10	-	-	-	-	-	-
Yes	1194	99	19	-	-	-	-	-	-
**†Feeding type**									
Exclusive breast feeding	1133	94	22	1	-	-	1	-	-
Replacement feeding	10	1	0	-	-	-	-	-	-
Ever mixed breast feeding	61	5	8	7.62	3.24–17.9	< 0.001	3.89	0.92–16.50	0.06
**Prophylaxis (baby)**									
No	14	1	8	-	-	-	-	-	-
Yes	1190	99	22	-	-	-	-	-	-
**†ART regiment (mother)**									
Tenofovir + Lamivudine + Efavirenz (Tenolam-E)	1151	96	16	1	-	-	1	-	-
None	29	2	11	43.35	17.7–106.40	< 0.001	-	-	-
Zidovudine (AZT)	24	2	3	10.13	2.74–37.43	0.001	0.17	0.02–1.46	0.11
**†Time ART treatment started**									
Before pregnancy	289	-	4	1	-	-	1	-	-
During pregnancy	841	-	13	1.11	0.36–3.46	0.85	1.01	0.31–3.32	0.99
After pregnancy	38	3	2	3.96	0.70–22.38	0.12	3.18	0.42–23.94	0.07
Unknown	36	3	0	-	-	-	-	-	-

ART, Antiretroviral treatment.

† , Variables entered into the multiple logistic regression model.

Of the 1204 infants, 30 (2.5% [95% CI, 1.7–3.5]) were HIV-infected. Twenty-two (2%) of 1133 infants who were exclusively breastfeeding and eight (13.11%) of 61 infants who were mixed feeding were HIV-infected. About 11 (22%) of 61 of the women practising mixed feeding had poor adherence to ART. None of the 10 formula-fed infants had a positive HIV DNA PCR test at 6 weeks of life. The majority (1166; 97%) of the women had vaginal delivery. Of the infants born via normal delivery, 2.5% were HIV-infected, and one (2.6%) of 38 infants (2.6%) born via Caesarean section was HIV-infected. The women were either on cART 1151 (96%), Zidovudine only 24 (2%) or not on ART 29 (2%). A higher proportion of infants of mothers not on ART (38%) and those on Zidovudine (13%) were HIV-infected compared with infants of mothers on cART (1%). Levels of HIV infection were infants whose mothers started ART before pregnancy (4 of 289; 1.4%) and those whose mothers started treatment during pregnancy (13 of 841; 1.5%) compared with infants whose mothers started ART after delivery (2 of 38; 5.3%) and those whose mothers were not on ART (11 of 36; 30.5%).

Adherence to ART reduced the odds of HIV infection by about 99% (odds ratio [OR] 0.01 [95% CI, 0.00–0.06]). Infants of mothers who started ART after delivery were three times as likely to be infected compared with those of mothers who started ART before pregnancy. The adjusted odds ratios for mothers who were on Zidovudine and for those mothers who started ART after delivery did not reach statistical significance. Mixed feeding had a borderline quadrupled odds of HIV infection compared with exclusive breastfeeding with a large confidence interval (OR 3.89 [95% CI, 0.92–16.50]) (see Table 1).

## Discussion

In this population of women living with HIV, of whom 96% accessed cART, we found a very low overall MTCT rate at 6 weeks of life. Combination antiretroviral treatment prior to conception or in pregnancy, cART adherence and exclusive formula feeding were associated with a low risk of HIV acquisition.

We also found that most (71%) of the women started cART during pregnancy. However, there were a few women (3%) who started cART after delivery, the infants of these women had a non-significant threefold odds of HIV infection compared with infants of women who started treatment before pregnancy. A larger sample size may have resulted in statistical significance. Late initiation of maternal ART was previously shown to be associated with the vertical transmission of HIV and infant mortality.^21,22,23,24^ It is unclear why these women were initiated on treatment lately. One possible reason could be that these women may have presented at the primary healthcare centre later in pregnancy, when they were in labour.^25^ A few women (2%) were not on ART. While these findings are encouraging, the Zimbabwe PMTCT programme needs to put more effort on having all women on ART soon after HIV diagnosis. These efforts are likely to further reduce the 6-week MTCT of HIV.

The current WHO infant feeding guidelines recommend breastfeeding even in the presence of mixed feeding.^14^ This recommendation was based on limited information on whether mixed feeding remains a risk for postnatal HIV transmission even in the presence of cART.^26,27^ Mixed-fed infants in our study had a statistically insignificant, but clinically important higher odds of HIV infection than exclusively breastfed infants, suggesting that the possibility of mixed feeding remains a risk even in the presence of cART. A large study would probably have achieved statistical significance. It is of concern that close to a quarter of the women practising mixed feeding had poor adherence to ART. Mixed feeding should therefore remain a concern for PMTCT programmes, and efforts should be made to promote and support exclusive breastfeeding for HIV-exposed infants.

Our study had several limitations. We used routinely collected data, and this limited us in determining the number of HIV infections that occurred during pregnancy and delivery and those that occurred through breastfeeding. We could not assess other factors that could influence the risk of HIV infection such as maternal viral load, or maternal age as they were not available in the reviewed clinical records. We did not account for ‘within clinic clustering’, which could influence the outcome. Our results may not be generalisable to infants who are premature and sick neonates who are at higher risk for HIV transmission.

## Conclusion

Despite the limitations of our study, the findings suggest that cART from conception or when initiated during pregnancy reduces the MTCT of HIV to levels similar to those found in controlled trial settings. Exclusive breastfeeding remains important even in the presence of ART.
